# Genomic epidemiological analysis of *mcr-1*-harboring *Escherichia coli* collected from livestock settings in Vietnam

**DOI:** 10.3389/fvets.2022.1034610

**Published:** 2022-10-26

**Authors:** Phuong Thi Lan Nguyen, Thi Hong Hanh Ngo, Thi Mai Hung Tran, Thi Ngoc Bich Vu, Viet Thanh Le, Hai Anh Tran, Duy Thai Pham, Ha Thanh Nguyen, Dieu Linh Tran, Thi Phuong Lien Nguyen, Thi Thi Tho Nguyen, Nhu Duong Tran, Duc Anh Dang, Anne-Laure Bañuls, Marc Choisy, H. Rogier van Doorn, Masato Suzuki, Huy Hoang Tran

**Affiliations:** ^1^National Institute of Hygiene and Epidemiology, Hanoi, Vietnam; ^2^Oxford University Clinical Research Unit, Hanoi, Vietnam; ^3^Quadram Institute Bioscience, Norwich Research Park, Norwich, United Kingdom; ^4^Hanoi Medical University, Hanoi, Vietnam; ^5^MIVEGEC (IRD-CNRS-Université de Montpellier), LMI DRISA, Center IRD, Montpellier, France; ^6^Oxford University Clinical Research Unit, Ho Chi Minh City, Vietnam; ^7^Centre for Tropical Medicine and Global Health, Nuffield Department of Clinical Medicine, University of Oxford, Oxford, United Kingdom; ^8^National Institute of Infectious Diseases, Tokyo, Japan

**Keywords:** *mcr-1*, *E. coli*, resistance, livestock, farm, plasmid harboring *mcr-1*, colistin resistance, antimicrobial resistance

## Abstract

Livestock has been implicated as a reservoir for antimicrobial resistance (AMR) genes that can spread to humans when antimicrobials are used in animals for food production to treat clinical diseases and prevent and control common disease events. In Vietnam, *mcr-1*-harboring *Escherichia coli* (MCRPEC) strains have been isolated from humans, animals (chickens, pigs, and dogs) feces, flies, foods, and the environment (rainwater, well water, and irrigation water) in communities and from clinical specimens in hospitals. The relationship between levels of AMR in livestock and its occurrence in humans is complex and is driven by many factors. We conducted whole genome sequencing of MCRPEC to analyze the molecular epidemiological characteristics, history, and relatedness of 50 isolates obtained in 2019 from different reservoirs in farms and markets in Ha Nam province, Vietnam. 34 sequence types (STs) with 3 new STs were identified in multilocus sequence typing analysis: ST12945 and ST12946 from chicken feces, and ST12947 from flies. The AMR phenotypes of 50 MCRPEC isolates were as follows: ampicillin (100%, 50/50), cefotaxime (10%, 5/50), gentamicin (60%, 30/50), amikacin (8%, 4/50), meropenem (6%, 3/50), ceftazidime (18%, 9/50), colistin (24%, 12/50) and ciprofloxacin (80%, 40/50). All 50 MCRPEC isolates were identified as MDR. 100% (50/50) isolates carried AMR genes, ranging from 5 to 22 genes. The most prevalent plasmid replicon types carrying *mcr-1* were IncP-1 (17/37, 45.9%), IncX4 (7/37, 18.9%), and IncHI2/IncHI2A (6/37, 16.2%). These data suggest that the epidemiology of the *mcr-1* gene is mostly determined by plasmid spreading instead of clonal dissemination of MCRPE strains. The co-occurrence of several STs such as ST10, ST48, ST155, ST206, ST2705 in various sample types, joined to the higher prevalence of a few types of Inc plasmids, confirms the dissemination of the *mcr-1* carrying plasmids in *E. coli* clones established in livestock. 5 over 8 STs identified in flies (ST206, ST2705, ST155, ST10, and ST48) suggested the fly contribution in the transmission of AMR bacteria in environments. These popular STs also occur in human samples and 100% of the human samples were positive for the *mcr-1* gene.

## Introduction

Antimicrobial resistance (AMR) is a global public health and development threat. In 2019, WHO declared that AMR has been one of the top ten global health threats facing humanity that caused least 700,000 deaths globally per year, which could potentially increase to 10 million deaths globally per year by 2050 (1, 2). The WHO Global Antimicrobial Resistance and Use Surveillance System (GLASS), 2021 emphasized high proportions of AMR in the bloodstream, and urinary and gastroenteric infections in most countries, including in last resort antimicrobials such as carbapenem, or first-line drugs such as co-trimoxazole (3). Livestock has been implicated as a reservoir for AMR that can spread to humans when antimicrobials are used in food animals to treat clinical diseases, prevent common disease events, and enhance animal growth, and many of these antimicrobials are identical to or closely resemble drugs used in humans (4, 5).

Carbapenem-resistant *Enterobacterales* (CRE) have spread worldwide and present a serious public health threat with high mortality rates as high resistance also occurs to other clinically important antimicrobials, such as cephalosporins, fluoroquinolones, and aminoglycosides (6). Colistin (Polymyxin E) is an old antimicrobial with cationic detergent properties that is considered to be a last therapeutic option to treat infections caused by carbapenem-resistant Gram-negative bacteria that are also resistant to all other first and second line drugs, because (i) the treatment options for first and second line drug resistant CRE remain very limited and (ii) colistin has proved its function in killing Gram-negative bacteria in severe infections (7–9). Colistin also has been used in veterinary medicine for decades, mainly for the prevention and treatment of *Enterobacterales* infection (10). While the spread of carbapenemase genes has been mainly recognized in humans but rarely evidenced in animals, colistin resistance in *Escherichia coli* seems to be related to the use of colistin in veterinary medicine on a global scale (11). The cases of colistin-resistant bacteria were reported from various parts of the world and became a major public health threat with the variation of genes encoding colistin resistance (*mcr-1* to *mcr-10*), especially the *mcr-1* gene that spread rapidly in animals, travelers, foods, and environment around the world (12, 13).

In Vietnam, MCRPEC strains have been isolated from humans, animals (chickens, pigs, and dogs), flies, foods, and the environment (rain, well, and irrigation water) in a community and from clinical specimens in referral hospitals (14–16). However, the relationship between levels of AMR in livestock and its occurrence in humans is complex and multi-factorial and includes the combinations of antimicrobials, bacterial strains, mobile genetic elements (MGEs), and livestock species, each with their dynamics (4, 17). The application of whole genome sequencing (WGS) as a potential powerful tool facilitates our baseline understanding of these pathways. Therefore, we conducted WGS to analyze molecular epidemiological characteristics, history, and relatedness of isolates obtained from different reservoirs in livestock settings.

## Materials and methods

### Bacterial isolates

Samples were obtained in 2019 from various sources in Ha Nam, a province in Northern Vietnam including poultry and swine farms, farmers and markets. Samples including farmer feces (20), chicken feces (61), pigs feces (31), dogs feces (18), farm flies (36), wastewater (21), and market surface (15) were collected and kept at – 80°C in the Antimicrobial Resistance Laboratory of the National Institute of Hygiene and Epidemiology (NIHE) in Vietnam. All samples were pre-processed as previously described in 2022 (16).

To screen for samples containing *mcr-1* genes, the samples were kept overnight in tryptic soy broth at 37°C without shaking. We used 1.5 ml of enriched broth for total DNA extraction and detected *mcr-1* by using PCR. Among *mcr-1* positive samples, we randomly selected 50% of the samples for further analysis.

The selected samples were cultured on MacConkey agar containing 0.5 mg/L colistin. We then picked up one *E. coli*-like colony represent for each sample for identification by MALDI-TOF MS (Bruker, Berlin, Germany). All confirmed *E. coli* isolates were selected for further screening for *mcr-1*. Next, DNA from *E. coli* isolates was subjected to confirm the presence of *mcr-1* by PCR.

Farm-scale is computed and classified by Decree 13/2020/ND-CP Detail Guideline of Livestock Law issued by the Vietnamese Government. Number of all types of food animals raised, including pigs, chickens, ducks, geese, quails, doves, ostriches, etc., is converted to “livestock unit” for each type of food animals, “total farm livestock unit” is sum of livestock units of all food animal types raised and is used to categorize farm-scale (House-hold: < 10 units, small: from 10 to under 30 units, medium: from 30 to under 300 units, large: ≥ 300 units) (18).

### Antimicrobial resistance phenotyping of MCRPEC isolates

The minimum inhibitory concentrations (MIC) to antimicrobials commonly used in treatment were determined on 50 MCRPEC isolates to identify susceptible (S) or resistant (R) phenotypes. The MCRPECs were tested for resistance against 8 drugs of 6 class of antimicrobials. MICs were determined using the agar dilution method for ampicillin (AMP), cefotaxime (CTX), gentamicin (GEN), amikacin (AMK), meropenem (MER), ceftazidime (CAZ), ciprofloxacin (CIP) and the microdilution method for colistin (CST) (Sigma-Aldrich) according to CLSI 2018 and European Committee on Antimicrobial Susceptibility Testing (EUCAST) 2018 (19, 20). *E. coli* ATCC 25922 and *Pseudomonas aeruginosa* ATCC 27853 were used as controls.

### Whole genome sequencing and genetic environment characterization of *mcr-1* genes

The procedure was performed as previously reported (14). Whole genome sequencing was performed on DNA from all *mcr-1*-positive *E. coli* isolates. Genomic DNA libraries of selected strains were prepared for WGS using the Nextera XT DNA Library Preparation Kit (Illumina), following the manufacturer's instructions. 300 bp paired-end sequencing was performed on an Illumina MiSeq platform (MiSeq Reagent Kit v3; 600 cycles). Raw sequence reads were *de novo* assembled into contigs using Shovill v1.1.0 with SPAdes v3.14.1 as the assembler. NCBI Prokaryotic Genome Annotation Pipeline (PGAP) was used to annotate genes. AMR genes from ResFinder databases of the Center for Genomic Epidemiology were identified using ABricate and Staramr (scans genome contigs against ResFinder. PlasmidFinder databases, https://libraries.io/pypi/staramr) were used to identify replicon typess, and MLST following Achtman sequence definitions database (https://pubmlst.org/bigsdb?db=pubmlst_ecoli_achtman_seqdef) was used to identify sequence type for the isolates. A maximum-likelihood phylogenetic trees based on the core genome single nucleotide polymorphisms (SNPs) were constructed from WGS data of the 50 MCRPEC isolates utilizing Prokka and Roary and IQ-TREE v1.6.11.

#### Phylogenetic groups

All isolates were assigned to phylogenetic groups (A, B1, C, B2, D, E and F) based on Clermont phylogenetic typing schemes (21).

#### Genes of virulence factors

Virulence genes were identified by VirulenceFinder V2.0 (https://cge.cbs.dtu.dk/services/VirulenceFinder/).

### Statistical methods

Isolates data were analyzed in MS Excel 2017 (Microsoft Corp., USA) using descriptive statistics as appropriate.

### Institutional review board statement

The study was conducted according to the guidelines of the Declaration of Helsinki and approved by the Institutional Review Board of the National Institute of Hygiene and Epidemiology, Vietnam (IRB approval number: HDDD−06/2019).

### Informed consent statement

Informed consent was obtained from all human subjects involved in the study.

## Results

### Samples containing *mcr-1* gene in livestock setting and *E. coli* isolates for whole genome sequencing

We found that 51.5% (104/202) of samples were *mcr-1* positive: 100% (20/20) of farmer feces samples, 62.3% (38/61) of chicken feces samples, 41.9% (13/31) of pig feces samples, 11.1% (2/18) of dog feces samples, 55.6% (20/36) of farm fly samples, 22.6% (7/31) of wastewater samples, and 26.7% (4/15) of market surface samples. We selected 50% (*n* = 52) from 104 *mcr-1* positive samples for further analysis. Among 52 selected samples, 50 samples appeared *E. coli* like colonies and all of them were confirmed *E. coli* by MALDI-TOF-Biotyper. Next, DNA of *E. coli* isolates was subjected to confirm the presence of *mcr-1* by PCR. Whole genome sequencing was performed on DNA from all *mcr-1*-positive *E. coli* isolates (*n* = 50) which included feces from farmer feces (*n* = 17) chicken feces (14), pig feces (4), dog feces (1), farm flies (11), wastewater (2), and market surface (1).

### Antimicrobial resistance phenotyping of MCRPEC isolates

Examining antimicrobial resistance of MCRPEC isolates, per drug and sample type, we observed that the resistance proportions to tested antimicrobials in the isolates were as follows: 100% (50/50) resistance against ampicillin, 10% (5/50) against cefotaxime, 60% (30/50) against gentamicin, 8% (4/50) against amikacin, 18% (9/50) against ceftazidime, 24% (12/50) against colistin, and 80% (40/50) against ciprofloxacin (Table 1). In particular, 6% (3/50) of isolates were at intermediate level with meropenem at MIC of 2 mg/L in comparison to breakpoint (CLSI 2019), indicating meropenem susceptibility reduction.

**Table 1:** Prevalence of antimicrobial resistance phenotype in MCRPEC isolates.

**Sample type**	**Investigated isolates**		**Resistance to investigating antimicrobials**															
			**AMP**		**CTX**		**GEN**		**AMK**		**MER**		**CAZ**		**CST**		**CIP**	
	**n**	**%**	**n**	**%**	**n**	**%**	**n**	**%**	**n**	**%**	**n**	**%**	**n**	**%**	**n**	**%**	**n**	**%**
Human feces	17	34.0	17	100.0	1	5.9	6	35.3	–	–	–	–	3	17.6	1	5.9	12	70.6
Chicken feces	14	28.0	14	100.0	3	21.4	9	64.3	3	21.4	2	14.3	4	28.6	1	7.1	11	78.6
Pig feces	4	8.0	4	100.0	–	–	3	75.0	–	–	–	–	1	25.0	2	50.0	4	100.0
Dog feces	1	2.0	1	100.0	–	–	1	100.0	–	–	–	–	–	–	–	–	1	100.0
Farm flies	11	22.0	11	100.0	–	–	9	81.8	–	–	–	–	–	–	6	54.5	9	81.8
Wastewater	2	4.0	2	100.0	–	–	1	50.0	–	–	–	–	–	–	1	50.0	2	100.0
Market surface	1	2.0	1	100.0	1	100.0	1	100.0	1	100.0	1	100.0	1	100.0	1	100.0	1	100.0
Total	50	100.0	50	100.0	5	10.0	31	62.0	4	8.0	3	6.0	9	18.0	12	24.0	40	80.0

Households which provided 78% (39/50) MCRPECs have resistance to more antimicrobials with 8 chosen antimicrobials than 18% (9/50) strains from medium-scale farms, 2% (1/50) strains from small-scale farms, and 2% (1/50) strains from market. 46% (23/50) and 44% (22/20) MCRPECs in this study belonged to poultry and mixed farms. Notably, there was one MCRPEC strain collected on the surface market but resistant to 8/8 antimicrobials tested in this study.

**Figure 1:**
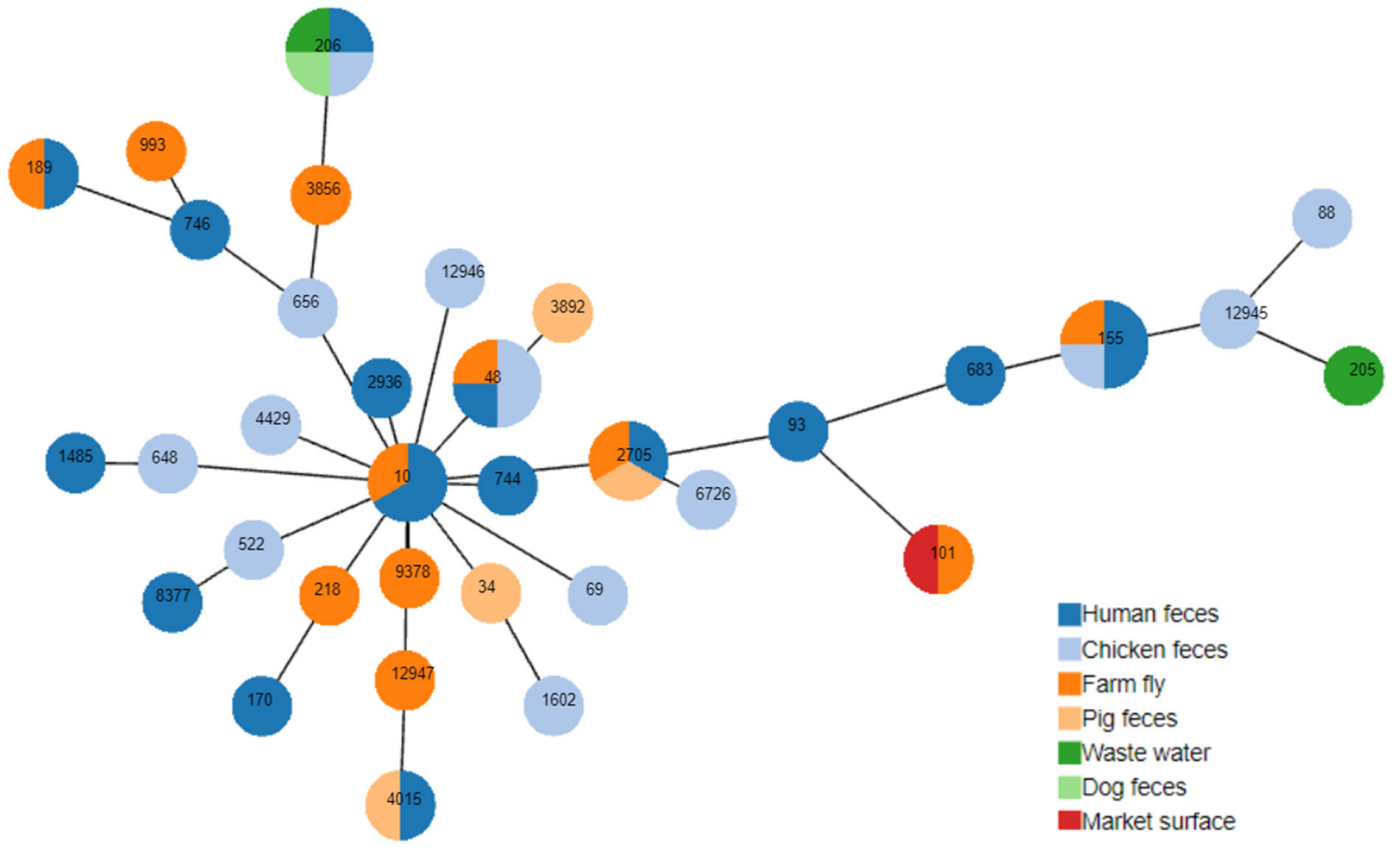
Minimum spanning tree based on multi-locus sequence types and seven housekeeping gene alleles of MCRPEC. Each circle corresponds to an individual sequence type (ST), the circle size indicated the number of isolates assigned to the same ST; The color of the circle denotes the sample type. The length of the branch is equal to the number of different alleles (calculated using seven MLST genes) between two linked nodes/STs.

Based on the PCR screening results, WGS was conducted to analyze AMR genes for further characterization of these species. The results of the MIC tests and WGS analysis are presented in Supplementary Table 1 and Figure 1. The AMR isolates showed strong resistance with MIC values higher than the breakpoint of resistance in recommendation of CLSI, such as 98% of strains were resistant to ampicillin at the MIC value >256 mg/L, 80% to cefotaxim (>64mg/L), and 75% to amikacin (>256 mg/L), respectively.

In our study, all isolates bearing *mcr-1* but 76% (38/50 of MCRPECs isolates showed susceptibility to colistin at MIC values ≤2 mg/L, of which 74% (37/50) strains had MIC value at 2 mg/L. Among which 24% (12/50) of isolates were resistant to colistin, of which 33% (4/12) were resistant at an MIC value >16 mg/L. Especially, three isolates showed reduced susceptibility to meropenem (3/50) at 2 mg/L.

Results from whole genome sequencing showed that 100% (50/50) of isolates harbored antimicrobial resistance genes (ARGs) with the range from 5 to 22 genes responsibility for resistance to colistin (*mcr-1*), aminoglycosides (*aac(3)-, aac(6')-, ant(3”)-IIa, aph(3”)-Ib, aph(3')-Ia, aph(6)-Id, aadA*), fosfomycin (*fosA*), β-lactams (*bla*_CARB − 3_, *bla*_CARB − 2_, *bla*_CTX − M_, *Escherichia*_*coli*_*ampC, Escherichia*_*coli*_*ampC*1_*beta*−*lactamase, bla*_OXA_, and *bla*_TEM − 1_), sulfonamides (*sul1, sul2, sul3*), fluoroquinolones (*qnrD1, qnrS*), tetracyclins [*tet(A)*], trimethoprim (*dfrA*), phenicols (*floR, catA4, catB3*), and macrolides (*emrE, mphA, mphB*) (Supplementary Table 1).

### MLST analysis and phylogenic tree

Thirty four sequence types (ST) of 50 selected MCRPECs were identified (Figure 1) and classified in 4 phylogroups (A, B1, C, and F) (Supplementary Table 1). Phylotyping analysis revealed that most of these strains belonged to the A group 38/50 (76%) with 26 different STs (ST10, ST34, ST48, ST69, ST93, ST101, ST155, ST170, ST189, ST205, ST206, ST648, ST656, ST683, ST744, ST746, ST1485, ST1602, ST2705, ST2936, ST3856, ST4015, ST6726, ST8377, ST12945, ST12946, ST12947, ST218, ST3892, ST4429, ST522, ST88, ST9378, and ST993). 9 / 50 strains (18%) were assigned to group B1 with 5 STs (ST101, ST155, ST205, ST683, and ST12945), 2/50 (4%) to group F with 2 STs (ST648, and ST1485) and 1 strain, with ST88, to group C. The eight most common STs were ST155 (*n* = 4; 8%); ST206 (*n* = 4; 8%), ST48 (*n* = 4; 8%), ST2705 (*n* = 3; 6%), ST10 (*n* = 3; 6%), ST 4015 (*n* = 2; 4%), ST101 (*n* = 2; 4%) and ST189 (*n* = 2; 4%). The minimum spanning tree based on multi-locus sequence types (Figure 1) showed the most of the STs found in this study belonged to clonal complex 10 (CC10). Three new STs were identified in this study, including ST12945, ST12946, and ST12947 with ST12945 collected from chicken feces and belonged to the phylogroup B1, ST12946 from chicken feces and ST12947 from flies belonged to the phylogroup A. There were several STs isolated from different sample types such as ST155 appeared on human feces, chicken feces, and farm flies; and ST2705 were obtained from human, pig feces, and farm flies. In addition, 5/8 STs from farm flies (ST206, ST2705, ST155, ST10, and ST48) and belonged to the A and B1 groups were also recorded on other sample types including humans. The co-occurrence of several STs in various sample types, joined to the higher prevalence of a few types of Inc plasmids, confirms the dissemination of the *mcr-1* carrying plasmids in *E. coli* clones established in livestock.

**Figure 2:**
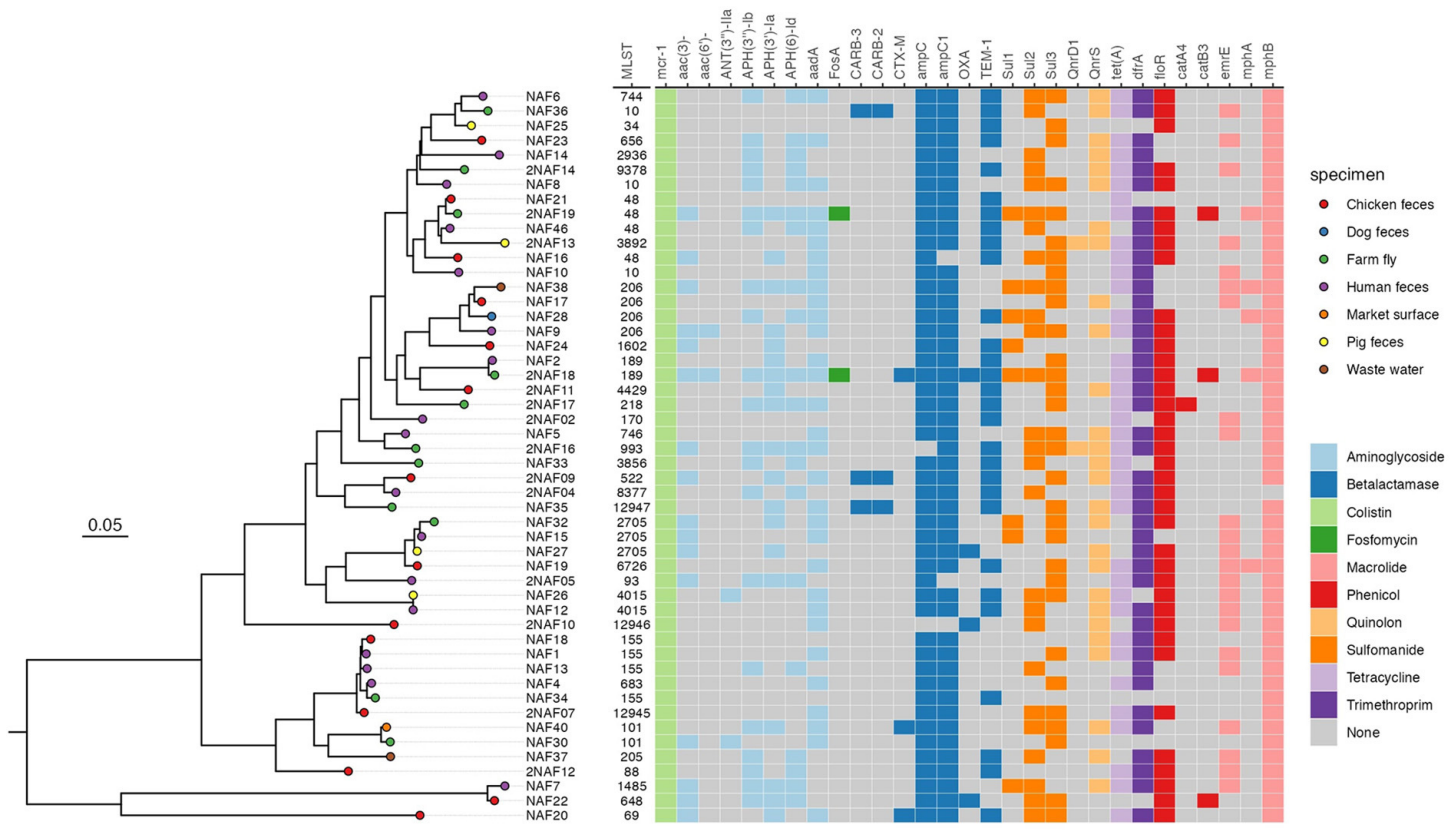
Maximum likelihood phylogenic analysis of 50 MCRPEC isolates from farms in Ha Nam. The heat map represents the AMR genotype of 50 MCRPECs.

The phylogenetic tree indicated two major clades: one clade (Figure 2) contained 47 isolates and 31 STs, including common STs like ST155, ST2705, ST48, ST4015, ST10, and ST206; the other clade (Figure 2) contained 3 isolates and 3 STs including ST69, ST648, and ST1485.

The core gene phylogeny revealed the close relation among MCRPECs isolated from different sample types and sources: ST2705, ST155, ST683, and ST206 have the close relation; ST2705 isolated from human, pig feces and flies, ST155 and ST683 from human, chicken feces, flies, ST206 from human, dog feces, market, wastewater.

All 50 MCRPECs harbored genes conferring resistance to various other antimicrobials: aminoglycosides, β-lactams, fosfomycin, macrolides, phenicols, fluoroquinolones, sulfonamide, tetracyclin, trimethoprim also presented on the Figure 2. Only three isolates (ST189, ST101, and ST69) harbored CTX-M gene encoding extended-spectrum beta-lactamase. Two of these isolates were collected from farm fly samples.

### Virulence factors genes

A total of 59 different virulence genes were detected from the MCRPECs. The virulence genes; *csgG, entA, entE, entS, fepC, fepD, fes* and *ompA* were detected in ≥ 98% the MCRPEC isolates. Other virulent genes that were commonly prevalent include; *aslA, csgB, csgD, csgF, entB, entC, entD, entF, fdeC, fepA, fepB, fepG, fimA, fimB, fimC, fimD, fimE, fimF, fimG, fimH, fimI, gspC, gspD, gspE, gspF, gspG, gspH, gspI, gspJ, gspK, gspL, gspM, yagV/ecpE, yagW/ecpD, yagX/ecpC, yagY/ecpB, yagZ/ecpA*, and *ykgk/ecpR*. The less prevalent (≤ 30%) were *astA, chuS, chuT, chuU, chuV, chuW, chuY, fyuA, iroN, iutA, kpsD, kpsM, and PapC*. The proportion of virulence genes from humans, animals, flies and environment was presented in Supplementary Tables 2, 3.

### Plasmid genotyping

**Figure 3:**
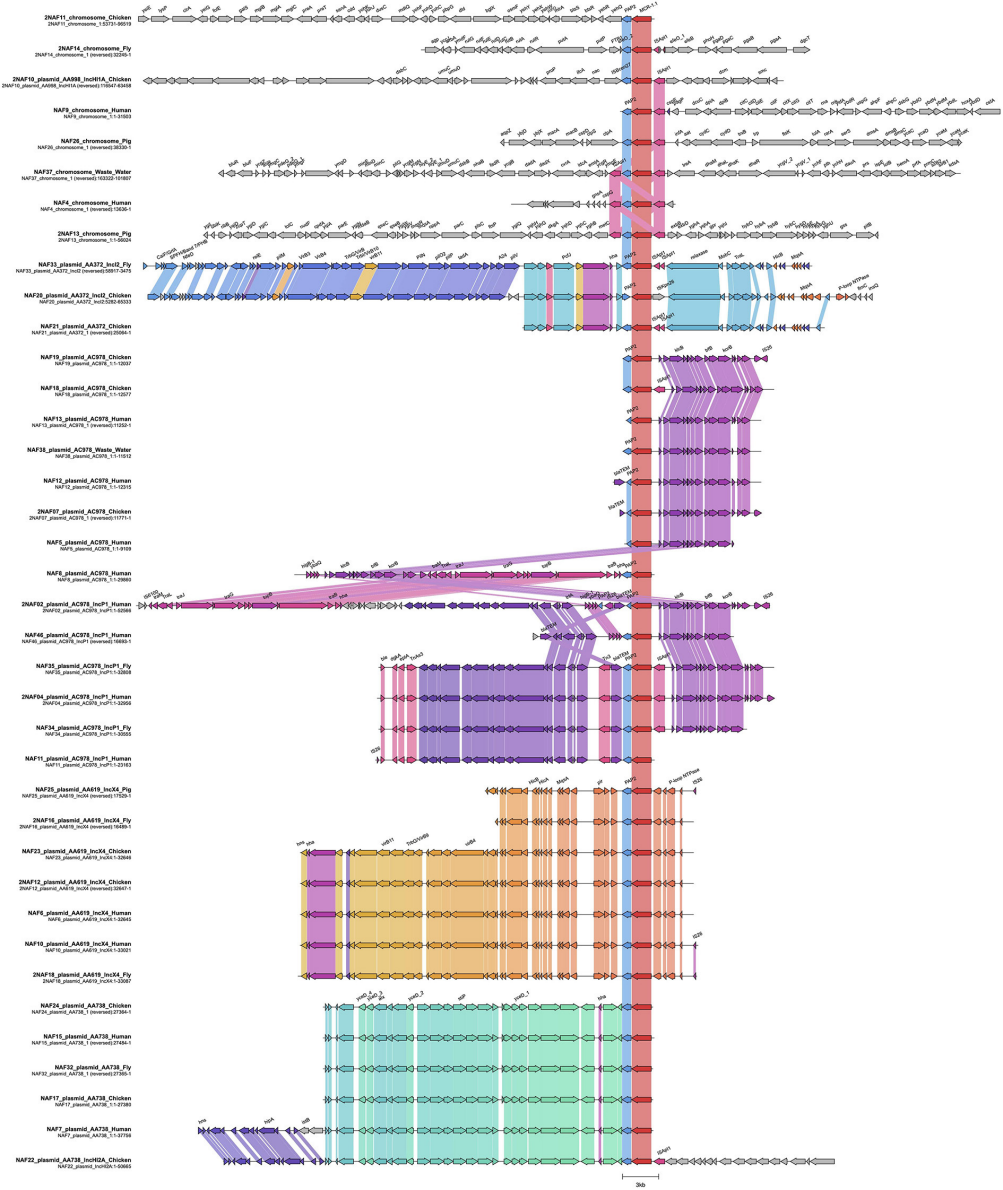
Representative of sequence alignment of *mcr-1*-containing cassettes. Arrow indicates an open reading frame and the direction of transcription. *ISApl1* is indicated by the pink arrow, *mcr-1* is indicated by the red arrow, the hypothetical gene is indicated by the gray arrow, and intrinsic genes of the plasmid backbone or chromosome are indicated by orange arrows. Color shadings connect regions of >99% similarity.

To classify the locations of *mcr-1* in the genomes, *mcr-1*-containing contigs were extracted from the complete genomes of MCRPEC isolates. We detected that *mcr-1* is located on both the chromosome (*n* = 7) and on plasmids (*n* = 43) isolates (Figure 3). Among plasmids carrying *mcr-1*, we identified 13 plasmid replicon types, the predominant plasmid replicon type was IncP-1 (17/37, 45.9%) followed by IncX4 (7/37, 18.9%), and IncHI2/IncHI2A (6/37, 16.2%). Other replicons were IncHI2/IncHI2A/IncN/p0111 (2/37, 5.4%), IncFIA(HI1)/IncHI1A/IncHI1B(R27) (1/37, 2.7%), IncHI2/IncHI2A/IncN (1/37, 2.7%), IncFIB(AP001918)/IncHI2/IncHI2A (1/37, 2.7%), p0111 (1/37, 2.7%), and IncY (1/37, 2.7%). Plasmid replicons of six isolates were unable to identify.

We observed 15 different genetic contexts of *mcr-1* with the presence of insertion sequence *ISApl1* on both chromosomes (*n* = 7) and plasmids (*n* = 8). Among 7 isolates carrying *mcr-1* on their chromosome, 3 contained the full *Tn6330* transposon sequence *ISApl1-pap2-mcr-1-ISApl1*. These strains were collected from human feces, pig feces and wastewater. Although, no full transposon was found on plasmids, 5 plasmids contained indication of the presence of *ISApl1* with a full copy downstream and 2 plasmids with a partial downstream *ISApl1* element. Among plasmids carrying the full copy of downstream *ISApl1*, 3 plasmids were IncP1 replicon, which has been known as an important plasmid type in antimicrobial transmission between gram negative bacteria.

## Discussion

Farms with high positive rate of *mcr-1* selected for sampling in this study had a high diversity of specimen types with MCRPECs; including fecal samples of humans and farm animals, such as chickens, dogs, fly samples, wastewater samples from farms and market surface samples. A study conducted in more than 25 nations on four continents also indicate the existence of *mcr-1* gene in animals, food, human, and the environment (22). Particularly, a study documented the rapid spread and proliferation of *E. coli* strains carrying the *mcr-1* gene in 2009, the first detection of *mcr-1*, in a retrospective study on *E. coli* strains isolated from chickens in the 1980s (23). In Vietnam, the prevalence of samples contain MCRPECs in this study was 51.5% (104/202), higher than the previous study in Ha Nam community in the Northern region between 2014 and 2015 with37.6% samples contain MCRPECs*E. coli* overall (14). A study conducted on chicken and pig farms in the Southern region showed that *E. coli* isolates had high levels of resistance (>20%) to important antimicrobials such as colistin (24). Meanwhile, another study in Thai Binh province reported that colistin-based drugs (CBD) were used in more than 50% of veterinary drugs purchased by chicken farms suggesting that CBD use could be responsible for increased resistance to colistin in extended spectrum β-lactamase-producing *E. coli* strains (25).

In 2017, the Vietnamese government issued the Circular 20/2017/TT-BNNPTNT that includes the instruction for controlling antimicrobials use in animal growth (26). However, the comparison between two studies illustrated that it will take more time for the rule to promote the efficiency in the Vietnamese livestock system by controlling and using antimicrobial properly.

In our study, MCRPECs isolates in our study originated from various sample resources, most of them were highly resistant to common antibiotics used in humans and agriculture, such as penicillin, cephalosporins, and aminoglycosides. However, most of them still showed susceptibility to meropenem, an antibiotic in the carbapenem family that is not permitted for use in livestock. There are three isolates that showed a reduced susceptibility to meropenem with a MIC value of 2 mg/L but no gene encoding carbapenemase enzyme [such as VIM-type, NDM-type, OXA-type and the serine carbapenemase genes (27)] was found in the whole genome sequencing analysis. It could be the result of an unknown gene conferring meropemen resistance, which will be revealed in future research.

The results of WGS confirmed this statement when they observed various AMR genes with a minimum of 5 resistance genes and a maximum of 22 AMR genes. *E. coli* is intrinsically susceptible to almost all clinically relevant antimicrobials, but this bacterium has a great capacity to accumulate resistance genes, primarily through horizontal gene transfer (11). 22 AMR genes found in 50 MCRPECs included colistin (*mcr-1*) and genes conferring resistant to aminoglycosides, fosfomycin, β-lactams, sulfonamides, fluoroquinolones, tetracyclins, trimethoprim, phenicols and macrolides, suggesting that the impact of MCRPECs on the transmission of AMR genes should not be underestimated.

Recent studies reported phenotypically susceptible or colistin-resistant MCRPECs strains at low MIC value (<6 mg/L) (28, 29). In this study, 76% (38/50 of MCRPECs strains showed susceptibility to colistin at MIC values ≤2 mg/L, 24% (12/50) showed colistin resistant phenotype, of which 33% (4/12) were resistant to colistin at high concentration (MIC >16 mg /L). In line with previous study conducted in Ha Nam in 2014–2015, the percentage of colistin-resistant MCRPECs with MIC value >6 mg /L in this study (4/50,8.0%) was <10% overall (14). However, our study reported similar prevalence of colistin-resistant *E. coli* strains as the study conducted in the Southern region of Vietnam, with colistin resistance rates in chickens and pigs of 22.2 and 24.4%, respectively (24). A recent study demonstrated that the acquisition of *mcr-1* by *E. coli* is a “poisoned chalice”. The high-level expression of *mcr-1* can reduce the growth rate of *E. coli* by compromising the bacterium's normal physiology, which has been observed in presence of selection pressure of colistin (30). Possibly the low proportion of high-level colistin resitance in MCRPECs indicates a low use of colistin in livestock setting but it permit for the omnipresence of *mcr-1* in various reservoirs. ADDIN EN.CITE (31, 32)Although it is insufficient evidence on the mechanism of interaction between the *mcr-1* gene and the colistin resistance of *E. coli*, the prevalence of MCRPECs with high MIC values in the agricultural farm environment exchangeability of genetic factors raise the alarm on the potential spread of drug-resistant strains in the community (31). This can lead to major challenges for public health systems in controlling the transmission of AMR strains of bacteria in the community.

Most of MCRPECs (74%) in this study had the MIC value at 2 mg/L coresponding with multiple reports of *mcr-1* positive *Enterobacteriaceae* with MIC values of ≤ 2 mg/L (32). Recent data on polymyxin pharmacokinetics (PK), pharmacodynamics (PD) have shown that a steady state concentration at 2 mg/L is required for killing bacteria with MICs of 2 mg/L. However, only nearly 50% of patients with normal renal function achieve this exposure and it is of limited clinical efficacy and is associated with substantial risk of nephrotoxicity (33). The omnipresence of *mcr-1* within Enterobacterales particularly in *E. coli*, which have been known opportunistic pathogens, thus could be a concern in clinical practice in future due to the effectiveness and safety at higher dose.

Previous study results suggested that there might be fly-mediated transmission of *mcr-1* from animals and the environment to humans, but there was no specific genetic evidence to verify this hypothesis (16). By sequencing technique, analyzing family trees, and analyzing multi-locus sequence types and seven housekeeping gene alleles of 50 strains of MCRPECs, our study brings support for this hypothesis through the presence of several clusters of STs on different reservoirs and the genetic similarity of STs isolated from flies to STs isolated from other reservoirs (ST206, ST2705, ST155, ST10, and ST48). Thus, it reflects that flies are vectors that are in the constant contact with various reservoirs of *E. coli*, and flies as such do not have a characteristic ST because they are transient hosts of *E. coli* by contact with other reservoirs (34). Alarmingly, 4/11 fly samples in this study carried colistin-resistant MCRPECs at MIC values >16 mg/L. There is not enough evidence to make the assertion about the role of flies in spreading of AMR genes in livestock environments but constant contact with various reservoirs of colistin-resistant MCRPECs should be consideration.

The study also showed a diversity of STs with 50 strains of MCRPECs showing 34 different STs from 4 phylo-groups (A, B1, C, F). Phylogenetic groups A (76%) and B1 (18%) were predominant among the tested isolates while group C and group F had low prevalence 2 and 4%, respectively. This result is in agreement with those reports that indicated *E. coli* from animal, human, environment, and food, mainly belonged to the A and B1 phylogenetic groups (35–37). Common STs found in this study, including ST48, ST2705, ST4015, ST206, ST10, ST189 (6STs belonging to group A), ST101, and ST155 (2 STs belonging to group B1) also appeared in many studies on food, livestock farms, and the environment in other countries, such as China, Poland, Korea, Laos, Thailand, France, Algeria (28, 38–41). Furthermore, the most of the STs found in this study belonged to clonal complex 10 (CC10), which has been known as the most dominant clonal group colonized in vertebrate hosts as a commensal in the gastrointestinal tract and aquatic environments, and carries diverse antimicrobial-resistance (AMR) genes and is known to cause extra-intestinal infections (42). It is also consistent with the major prevalence of *E. coli* ST10 worldwide (43). Three new STs discovered in this study were isolated from chicken feces samples (ST12495, ST12946) and fly samples (ST12947), the latter one being resistant to colistin at MIC value >16 mg/L. Among them, two STs (ST12946 and ST12947) belonged to CC10. The occurrence of STs in CC10 is likely result of genetic exchange among bacterial strains from animals, humans and the environment. This is also the model that contributes to the spread of resistance genes. In many livestock farms in Vietnam, antimicrobials are commonly used in the treatment or prevention of livestock diseases, or as growth promoters, including aminoglycosides, tetracyclines, phenicols, β-lactams, colistin, sulfonamides and fluoroquinolones groups (44, 45). This is considered a cause of increased AMR in the community including in humans, animals, and environment. A retrospective study conducted in Ha Nam, Vietnam, showed high prevalence of AMR genes with varied resistance data depending on the type of sample in humans, animals, and the environment (15). MCRPECs strains isolated in this study have a high proportion of carrying resistance genes in the groups of colistin, aminoglycosides, fosfomycin, β-lactams, sulfonamides, fluoroquinolones, tetracyclines, trimethoprim, phenicols, and macrolides, which is consistent with previous reports on AMR gene-carrying data in rural Vietnamese communities (15).

*E. coli* strains can possess different virulence markers to invade and colonize inner layers of the intestinal lining causing infections with different clinical symptoms (46). VFs include five main groups: (1) adhesions; (2) toxins; (3) siderophores; (4) capsule production and (5) protections and invasions (47). There was a strong link between the virulence and phylogeny in *E. coli* infections. Commensal *E. coli*, with no pathogenic features and occurring, among other places, on the gastrointestinal tract mucosa, most often belong to group A or B1. Pathogenic *E. coli* responsible for intestinal infections belong to phylogenetic groups A, B1 or D. *E. coli* responsible for extra-intestinal infections belong to groups B2 and D, group F is related to the main group B2 (21, 48). These isolates in our study harbor a total of 59 different virulence genes and classified in 4 phylogroups (A, B1, C, F). Our results show that MCRPEC strains isolated from livestock, farmers, flies, environment disposed of various virulence genes. Frequently recorded virulence genes comprised genes related to invasion and iron-uptake in *E. coli* (invasion-*aslA, ompA, kpsD, kpsM, ompA*; iron-uptake *chuS, chuT, chuU, chuV, chuW, chuY, entA, entB, entC, entE, entF, fepA, fepB, fepC, fepD, fepG* …), the less prevalent virulence genes was reported the close related to ESBL genes such as *iutA; fmH, papC* encode the adhesion subunit of type 1 fimbriae and related to colonization. Overal, the presence of MCRPECs bearing different virulence genes which are reported in extraintestinal pathogenic *E. coli* (ExPEC) (49). This can be considered a warning for the risk of infection by pathogenic strain from communities.

Studies on the *mcr-1* gene showed that there were many types of plasmids that could carry *mcr-1* belonging to incompatibility groups IncX4, IncI2, InHI2, IncF, IncFIB, IncY and IncP-1 (11, 50). In this research, up to 10 plasmids carrying *mcr-1* gene are reported, including common plasmids reported worldwide, in which the most prevalent is IncP-1 (17/37, 45.9%), followed by IncX4 (7/37, 18.9%). IncP-1 plasmid group is widely distributed in feces, soil, wastewater treatment plants and in both pathogenic and opportunistic species of bacteria, playing an important role in encoding multidrug resistance and bacterial adaptation by horizontal gene transfer (51). 45.9% of MCRPEC strains in this study have IncP-1 plasmid, which is a serious concern as IncP-1 plasmids can encode multidrug resistance, carry adaptive genes, AMR genes, virulence genes to migrate to new host through conjugated transfer and remains stable in bacterial cells (51). Therefore, we believe that in the future, deeper studies to determine the extent and risk of spreading resistance genes located on these plasmids are highly needed. Two major plasmids in our study, IncX4 (18.9%) and IncHI2/IncHI2A (16.2%) were also common in clinical intestinal bacteria strains associated with the prevalence of AMR genes (50, 52, 53). Thus, the AMR gene diversity and AMR phenotype of MCRPECs strains in this study could be the result of horizontal gene transfer from different strains on different hosts. These strains could become the next transmission threat due to broad-host range plasmids. A report from Americas also suggest that the epidemiology of the *mcr-1* gene in the Americas is mostly determined by plasmid spreading instead of clonal dissemination of MCRPE strains (43).

Recent studies have proposed that the *mcr-1* is mobilized by a composite transposon (*Tn6330)* flanked by two ISA*pl1* insertion sequences. In this study, we found full genetic context of the *Tn6330* transposon, which was found on only chromosome of three *E. coli* isolates. In line with previous studies, these findings suggested that the insertion of *mcr-1* into the chromosome of *E. coli* occurred recently. However, we also detected a loosening of ISA*pl1* insertion sequences on both chromosome and plasmid harboring *mcr-1*, indicating that the *mcr-1* is being stabilized on the core genetic backbone in the ecological niches (54).

This study revealed the high possibility of spreading drug resistance genes through the vector mediated by flies in livestock farms in Ha Nam. This process is occurring alarmingly in livestock environments with clear evidence, in which samples from humans, livestock, wastewater, and traditional markets obtained strains of MCRPEC that were resistant to many antimicrobials. In the studied strains, the appearance of new STs is a sign of continuous recombination process between bacterial strains from the community and the environment. The occurrence of *mcr-1* in different plasmids indicates high mobility of this gene in *E. coli* as a host. One limitation in this study is the lack of access to information on the use of antimicrobials in farms to be able to evaluate the impact of selective pressure of livestock antimicrobials on collected strains.

## Data availability statement

The data of this study have been deposited in the European Nucleotide Archive (ENA) at EMBL-EBI under accession number PRJEB55625 (https://www.ebi.ac.uk/ena/browser/view/PRJEB55625).

## Author contributions

HHT, HRvD, A-LB, DAD, and NDT design the study and directed study implementation. HHT, TMHT, PTLN, DTP, TTTN, and TPLN managed the fieldwork. HHT, HTN, and HAT performed laboratory work. PTLN, DTP, HTN, DLT, and THHN managed data. HHT, PTLN, VTL, THHN, TNBV, MC, and MS analyzed data. HHT, PTLN, TNBV, and THHN developed the first draft of the paper. All the authors reviewed and edited drafts of the manuscript and approved the final version.

## Funding

This study is funded by Vietnam National Foundation for Science and Technology Development (NAFOSTED) under grant number 108.02-2017.320, GCRF OneHealth Poultry Hub (Royal Veterinary College, UK) under grant number BB/S001269/1, the Japan Agency for Medical Research and Development (AMED) under grants numbers JP22gm1610003, JP22fk0108133, JP22fk0108139, JP22fk0108642, JP22wm0225004, JP22wm0225008, JP22wm0225022, JP22wm0325022, JP22wm0325003, and JP22wm0325037, and from IRD and LMI DRISA.

## Conflict of interest

The authors declare that the research was conducted in the absence of any commercial or financial relationships that could be construed as a potential conflict of interest.

## Publisher's note

All claims expressed in this article are solely those of the authors and do not necessarily represent those of their affiliated organizations, or those of the publisher, the editors and the reviewers. Any product that may be evaluated in this article, or claim that may be made by its manufacturer, is not guaranteed or endorsed by the publisher.
