# Supplementary material for: Genomic epidemiological analysis of mcr-1-harboring Escherichia coli collected from livestock settings in Vietnam
Source: Front Vet Sci. 2022 Oct 26;9:1034610. doi: 10.3389/fvets.2022.1034610 (PMC9643773; doi:10.3389/fvets.2022.1034610)
Supplement: Supplementary file 1 [file Table_1.DOCX]

Table S1. AMR genotypes, phenotypes, and virulence of MCRPEC isolates

| **No.** | **Sample ID** | **Phylo-groups** | **Plasmid type/ Chromosome carriage *mcr-1*** | **Sample type** | **ST** | **AMR genes** | **MIC (mg/L)** | | | | | | | |
| --- | --- | --- | --- | --- | --- | --- | --- | --- | --- | --- | --- | --- | --- | --- |
|  |  |  |  |  |  |  | **AMP** | **CTX** | **CAZ** | **CIP** | **GEN** | **MER** | **AMK** | **CST** |
| 1 | NAF1 | B1 | IncP-1 | Human feces | 155 | *mcr-1*, aadA, ampC, ampC1, Sul3, QnrS, tet(A), dfrA, floR, emrE, mphB | R  (>256) | S  (0.125) | S  (2) | R  (16) | S  (0.25) | S  (0.125) | S  (4) | S  (2) |
| 2 | NAF2 | A | Unknown plasmid | Human feces | 189 | *mcr-1*, APH(3'')-Ia, , aadA, ampC, ampC1, TEM-1, Sul3, tet(A), dfrA, floR, mphB | R  (>256) | S  (0.125) | S  (0.5) | S  (0.125) | S  (0.25) | S  (0.125) | S  (0.5) | S  (2) |
| 3 | NAF4 | B1 | *mcr-1* locates on chromosome | Human feces | 683 | *mcr-1*, aadA, ampC, ampC1, Sul3, tet(A), dfrA, mphB | R  (>256) | S  (0.125) | S  (1) | R  (8) | S  (0.25) | S  (0.125) | S  (1) | S  (2) |
| 4 | NAF5 | A | IncP-1 | Human feces | 746 | *mcr-1*, aadA, ampC, ampC1, Sul2, Sul3, QnrS, tet(A), dfrA, floR, emrE, mphB | R  (>256) | S  (0.125) | S  (1) | R  (16) | S  (0.25) | S  (0.125) | S  (1) | S  (2) |
| 5 | NAF6 | A | IncX4 | Human feces | 744 | *mcr-1*, APH(3'')-Ib, APH(6)-Id, aadA, ampC, ampC1, TEM-1, ul2, Sul3, QnrS, tet(A), dfrA, floR, mphB | R  (>256) | S  (0.125) | S  (1) | R  (8) | I  (8) | S  (0.125) | S  (8) | S  (2) |
| 6 | NAF7 | F | IncHI2, IncHI2A, IncN, p0111 | Human feces | 1485 | *mcr-1*, aac(3)-, APH(3'')-Ib, APH(3'')-Ia, APH(6)-Id, ampC, ampC1, Sul1, Sul2, QnrS, tet(A), dfrA, floR, emrE, mphB | R  (>256) | S  (0.125) | S  (1) | R  (16) | R  (32) | S  (0.125) | S  (1) | S  (2) |
| 7 | NAF8 | A | IncP-1 | Human feces | 10 | *mcr-1*, APH(3'')-Ib, APH(6)-Id, aadA, ampC, ampC1, Sul2, Sul3, QnrS, tet(A), dfrA, floR, mphB | R  (>256) | S  (0.125) | S  (2) | R  (32) | S  (0.25) | S  (0.125) | S  (8) | S  (2) |
| 8 | NAF9 | A | *mcr-1* locates on chromosome | Human feces | 206 | *mcr-1*, aac(3)-, aac(6')-, APH(3'')-Ia, , aadA, ampC, ampC1, Sul2, Sul3, QnrS, tet(A), dfrA, floR, mphB | R  (>256) | S  (0.125) | S  (1) | R  (16) | R  (32) | S  (0.125) | S  (2) | S  (2) |
| 9 | NAF10 | A | IncX4 | Human feces | 10 | *mcr-1*, aadA, ampC, ampC1, Sul3, tet(A), dfrA, emrE, mphB | R  (>256) | S  (0.125) | S  (0.25) | S  (0.125) | S  (0.5) | S  (0.25) | S  (1) | S  (2) |
| 10 | NAF12 | A | IncP-1 | Human feces | 4015 | *mcr-1*, aadA, ampC, ampC1, TEM-1, Sul2, QnrS, tet(A), dfrA, floR, emrE, mphB | R  (>256) | S  (0.125) | R  (>64) | I  (1) | S  (0.5) | S  (0.125) | S  (2) | S  (2) |
| 11 | NAF13 | B1 | IncP-1 | Human feces | 155 | *mcr-1*, APH(3'')-Ib, , APH(6)-Id, ampC, ampC1, Sul2, dfrA, emrE, mphB | R  (>256) | S  (0.125) | R  (>64) | I  (0.5) | R  (16) | S  (0.125) | S  (1) | S  (2) |
| 12 | NAF14 | A | IncP-1 | Human feces | 2936 | *mcr-1*, APH(3'')-Ib, APH(6)-Id, ampC, ampC1, Sul2, QnrS, tet(A), dfrA, mphB | R  (>256) | S  (0.125) | S  (1) | I  (0.5) | S  (0.5) | S  (0.125) | S  (2) | I  (4) |
| 13 | NAF15 | A | IncHI2, IncHI2A, IncN, p0111 | Human feces | 2705 | *mcr-1*, aac(3)-, aadA, ampC, ampC1, Sul1, Sul3,dfrA, emrE, mphB | R  (>256) | S  (0.5) | S  (4) | S  (0.06) | R  (64) | S  (0.125) | S  (0.5) | S  (2) |
| 14 | NAF16 | A | IncP-1 | Chicken feces | 48 | *mcr-1*, aac(3)-, APH(3'')-Ia, aadA, ampC, TEM-1, Sul2, Sul3, tet(A), dfrA, floR, mphB | R  (>256) | S  (0.125) | S  (1) | I  (1) | R  (32) | S  (0.25) | S  (1) | S  (2) |
| 15 | NAF17 | A | IncHI2, IncHI2A, IncN | Chicken feces | 206 | *mcr-1*, APH(3'')-Ib, APH(6)-Id, aadA, ampC, ampC1, TEM-1, Sul3, QnrS, tet(A), dfrA, emrE, mphB | R  (>256) | S  (0.125) | S  (2) | R  (32) | S  (0.5) | S  (0.125) | S  (4) | S  (1) |
| 16 | NAF18 | B1 | IncP-1 | Chicken feces | 155 | *mcr-1*, ampC, ampC1, TEM-1, Sul3, floR, mphB | R  (>256) | S  (0.125) | S  (1) | I  (1) | S  (4) | S  (0.125) | S  (1) | S  (2) |
| 17 | NAF19 | A | IncP-1 | Chicken feces | 6726 | *mcr-1*, aadA, ampC, ampC1, TEM-1, Sul3, QnrS, tet(A), dfrA, floR, emrE, mphA, mphB | R  (>256) | S  (0.125) | S  (2) | R  (2) | S  (0.5) | S  (0.125) | S  (1) | S  (2) |
| 18 | NAF20 | A | Unknown plasmid | Chicken feces | 69 | *mcr-1*, aac(3)-, APH(3'')-Ia, aadA, CTX-M, ampC, ampC1, TEM-1, Sul2, Sul3, tet(A), dfrA, floR, mphB | R  (>256) | R  (>64) | I  (8) | R  (>32) | R  (128) | S  (0.25) | S  (2) | S  (2) |
| 19 | NAF21 | A | Unknown plasmid | Chicken feces | 48 | *mcr-1*, ampC, ampC1, TEM-1, tet(A), mphB | R  (>256) | S  (0.125) | S  (1) | I  (1) | S  (0.5) | S  (0.125) | S  (1) | S  (2) |
| 20 | NAF22 | F | IncHI2, IncHI2A | Chicken feces | 648 | *mcr-1*, aac(3)-, APH(3'')-Ib, APH(3'')-Ia, APH(6)-Id, ampC, ampC1, OXA, Sul2, Sul3, floR, catB3, mphB | R  (>256) | S  (0.125) | R  (16) | R  (32) | R  (>128) | S  (0.125) | R  (256) | S  (2) |
| 21 | NAF23 | A | IncX4 | Chicken feces | 656 | *mcr-1*, APH(3'')-Ib, APH(6)-Id, aadA, ampC, ampC1, TEM-1, Sul3, QnrS, tet(A), dfrA, emrE, mphB | R  (>256) | S  (0.125) | S  (1) | R  (32) | R  (32) | S  (0.125) | S  (2) | S  (2) |
| 22 | NAF24 | A | IncHI2, IncHI2A | Chicken feces | 1602 | *mcr-1*, aac(3)-, APH(3'')-Ia, ampC, ampC1, TEM-1, Sul1, dfrA, floR, mphB | R  (>256) | S  (0.25) | S  (2) | S  (0.06) | R  (>128) | I  (2) | R  (>257) | S  (2) |
| 23 | NAF25 | A | IncX4 | Pig feces | 34 | *mcr-1*, ampC, ampC1, TEM-1, Sul3, floR, mphB | R  (>256) | S  (0.125) | S  (2) | R  (16) | S  (1) | S  (0.125) | S  (4) | S  (2) |
| 24 | NAF26 | A | *mcr-1* locates on chromosome | Pig feces | 4015 | *mcr-1*, ANT(3'')-IIa, aadA, ampC, ampC1, TEM-1, Sul2, Sul3, QnrS, tet(A),floR, emrE, mphB | R  (>256) | S  (0.125) | S  (1) | R  (2) | I  (8) | S  (0.125) | S  (1) | S  (2) |
| 25 | NAF27 | A | IncHI2, IncHI2A | Pig feces | 2705 | *mcr-1*, aac(3)-, APH(3'')-Ia, ampC, ampC1, OXA, QnrS, dfrA, floR, emrE,mphB | R  (>256) | S  (0.25) | I  (8) | I  (1) | R  (64) | S  (0.125) | S  (2) | I  (4) |
| 26 | NAF28 | A | Unknown plasmid | Dog feces | 206 | *mcr-1*, APH(3'')-Ib, APH(6)-Id, aadA, ampC, ampC1, TEM-1, Sul1, Sul2, tet(A), dfrA, floR, mphA, mphB | R  (>256) | S  (0.125) | S  (2) | R  (>32) | R  (16) | S  (0.125) | S  (2) | S  (2) |
| 27 | NAF30 | B1 | *mcr-1* locates on chromosome | Farm fly | 101 | *mcr-1*, aac(3)-, ANT(3'')-IIa, aadA, ampC, ampC1, Sul3, mphB | R  (>256) | S  (0.125) | S  (2) | R  (32) | R  (64) | S  (0.125) | S  (2) | S  (2) |
| 28 | NAF32 | A | IncHI2, IncHI2A | Farm fly | 2705 | *mcr-1*, aac(3)-, APH(3'')-Ia, , aadA, ampC, ampC1, Sul1, Sul3, QnrS, dfrA, floR, emrE, mphB | R  (>256) | S  (0.25) | S  (4) | I  (1) | R  (64) | S  (0.125) | S  (1) | S  (2) |
| 29 | NAF33 | A | Unknown plasmid | Farm fly | 3856 | *mcr-1*, APH(3'')-Ib, APH(6)-Id, ampC, ampC1, TEM-1, Sul2, QnrS, tet(A), floR, mphB | R  (>256) | S  (0.125) | S  (1) | I  (1) | I  (8) | S  (0.25) | S  (4) | I  (4) |
| 30 | NAF34 | B1 | IncP-1 | Farm fly | 155 | *mcr-1*, ampC, ampC1, TEM-1, mphB | R  (>256) | S  (0.125) | S  (1) | I  (1) | S  (0.5) | S  (0.125) | S  (1) | I  (4) |
| 31 | NAF35 | A | IncP-1 | Farm fly | 12947 | *mcr-1*, APH(3'')-Ia, aadA, CARB-3, CARB-2, ampC, ampC1, TEM-1, Sul3, QnrS, tet(A), dfrA, floR, mphB | R  (>256) | S  (0.125) | S  (0.5) | S  (0.125) | S  (0.5) | S  (0.5) | S  (1) | R  (>16) |
| 32 | NAF36 | A | IncP-1 | Farm fly | 10 | *mcr-1*, CARB-3, CARB-2, ampC, ampC1, TEM-1, Sul2, QnrS, tet(A), dfrA, floR, emrE, mphB | R  (>256) | S  (0.125) | S  (1) | I  (0.5) | R  (32) | S  (0.125) | S  (2) | R  (>16) |
| 33 | NAF37 | B1 | IncHI2, IncHI2A | Wastewater | 205 | *mcr-1*, APH(3'')-Ib, APH(6)-Id, ampC, ampC1, TEM-1, Sul2, QnrS, tet(A), dfrA, floR, emrE, mphB | R  (>256) | S  (0.125) | S  (4) | R  (2) | S  (2) | S  (0.125) | S  (1) | R  (8) |
| 34 | NAF38 | A | IncP-1 | Wastewater | 206 | *mcr-1*, aac(3)-, APH(3'')-Ib, APH(3'')-Ia, APH(6)-Id, aadA, ampC, ampC1, Sul1, Sul2, Sul3, tet(A), dfrA, emrE, mphA, mphB | R  (>256) | S  (0.125) | S  (2) | I  (1) | R  (16) | S  (0.25) | S  (1) | S  (2) |
| 35 | NAF40 | B1 | IncHI2, IncHI2A | Market surface | 101 | *mcr-1*, APH(3'')-Ib, APH(3'')-Ia, aadA, CTX-M, ampC, ampC1, Sul2, Sul3, QnrS, tet(A), dfrA, emrE, mphB | R  (>256) | R  (>64) | R  (>64) | I  (0.5) | R  (>128) | I  (2) | R  (>257) | I  (4) |
| 36 | NAF46 | A | IncP-1 | Human feces | 48 | *mcr-1*, APH(3'')-Ib, APH(6)-Id, aadA, ampC, ampC1, TEM-1, Sul2, QnrS, dfrA, floR, mphB | R  (128) | S  (0.125) | S  (1) | I  (0.5) | S  (0.5) | S  (0.125) | S  (1) | S  (2) |
| 37 | 2NAF02 | A | IncP-1 | Human feces | 170 | *mcr-1*, ampC, ampC1, TEM-1, tet(A), floR, emrE, mphB | R  (>256) | S  (0.125) | S  (1) | S  (0.06) | S  (0.25) | S  (0.25) | S  (1) | S  (2) |
| 38 | 2NAF04 | A | IncP-1 | Human feces | 8377 | *mcr-1*, APH(3'')-Ib, APH(6)-Id, ampC, ampC1, TEM-1, Sul2, tet(A), dfrA, floR | R  (>256) | S  (0.125) | S  (1) | S  (0.06) | S  (0.5) | S  (0.25) | S  (2) | S  (2) |
| 39 | 2NAF05 | A | IncFIB(AP001918), IncHI2, IncHI2A | Human feces | 93 | *mcr-1*, aac(3)-,APH(3'')-Ib, APH(3'')-Ia, APH(6)-Id, ampC, Sul3, tet(A), dfrA, floR, emrE, mphB | R  (>256) | R  (>64) | R  (>64) | I  (0.5) | R  (32) | S  (0.25) | S  (1) | S  (2) |
| 40 | 2NAF07 | B1 | IncP-1 | Chicken feces | 12945 | *mcr-1*, aadA, ampC, ampC1, Sul2, Sul3, tet(A), dfrA, floR, mphB | R  (>256) | S  (0.125) | S  (1) | R  (8) | S  (0.5) | S  (0.125) | S  (1) | S  (2) |
| 41 | 2NAF09 | A | p0111 | Chicken feces | 522 | *mcr-1*, aac(3)-, APH(3'')-Ia, , aadA, CARB-3, CARB-2, , ampC, ampC1, TEM-1, Sul3, QnrS, tet(A), dfrA, floR, mphB | R  (>256) | S  (0.125) | S  (2) | I  (1) | R  (32) | S  (0.125) | S  (1) | S  (2) |
| 42 | 2NAF10 | A | IncFIA(HI1), IncHI1A, IncHI1B(R27) | Chicken feces | 12946 | *mcr-1*, aadA, OXA, Sul2, QnrS, dfrA, floR, emrE, mphB | R  (>256) | S  (0.125) | S  (0.5) | S  (0.25) | R  (16) | S  (0.125) | S  (1) | S  (2) |
| 43 | 2NAF11 | A | *mcr-1* locates on chromosome | Chicken feces | 4429 | *mcr-1*, APH(3'')-Ia, ampC, ampC1, TEM-1, Sul3, QnrS, tet(A), dfrA, floR, mphB | R  (>256) | R  (32) | R  (64) | R  (32) | R  (16) | S  (0.125) | S  (2) | I  (4) |
| 44 | 2NAF12 | C | IncX4 | Chicken feces | 88 | *mcr-1*, APH(3'')-Ib, APH(6)-Id, ampC, ampC1, TEM-1, tet(A), dfrA, floR, emrE, mphB | R  (>256) | R  (>64) | R  (>64) | S  (0.25) | R  (>128) | I  (2) | R  (>257) | S  (2) |
| 45 | 2NAF13 | A | *mcr-1* locates on chromosome | Pig feces | 3892 | *mcr-1*, aadA, ampC, ampC1, TEM-1, Sul3, QnrD1, QnrS, tet(A), dfrA, floR, emrE, mphB | R  (>256) | S  (0.125) | S  (2) | R  (2) | R(32) | S  (0.25) | S  (1) | I  (4) |
| 46 | 2NAF14 | A | *mcr-1* locates on chromosome | Farm fly | 9378 | *mcr-1*, APH(3'')-Ib, , APH(6)-Id, ampC, ampC1, TEM-1, Sul2, QnrS, tet(A), dfrA, floR, emrE, mphB | R  (>256) | S  (0.125) | S  (1) | R  (16) | R  (16) | S  (0.125) | S  (2) | S  (2) |
| 47 | 2NAF16 | A | IncX4 | Farm fly | 993 | *mcr-1*, aac(3)-, APH(3'')-Ib, APH(3'')-Ia, APH(6)-Id, aadA, ampC1, TEM-1, Sul2, Sul3, QnrD1, QnrS, tet(A), dfrA, floR, mphB | R  (>256) | S  (0.125) | S  (1) | I  (1) | R  (32) | S  (0.125) | S  (1) | S  (2) |
| 48 | 2NAF17 | A | Unknown plasmid | Farm fly | 218 | *mcr-1*, APH(3'')-Ib, APH(3'')-Ia, APH(6)-Id, aadA, ampC, ampC1, TEM-1, Sul3, tet(A), dfrA, floR, catA4, mphB | R  (>256) | S  (0.25) | S  (2) | I  (0.5) | R  (16) | S  (0.25) | S  (2) | R  (>16) |
| 49 | 2NAF18 | A | IncX4 | Farm fly | 189 | *mcr-1*, aac(3)-, aac(6')-, APH(3'')-Ib, APH(3'')-Ia, APH(6)-Id, aadA, FosA, CTX-M, ampC, ampC1, OXA, TEM-1, Sul1, Sul2, Sul3, tet(A), dfrA, floR, catB3, mphA, mphB | R  (>256) | S  (0.125) | S  (1) | S  (0.125) | R  (128) | S  (0.125) | S  (1) | R  (>16) |
| 50 | 2NAF19 | A | IncY | Farm fly | 48 | *mcr-1*, aac(3)-, APH(3'')-Ib, APH(3'')-Ia, APH(6)-Id, aadA, FosA, ampC, ampC1, TEM-1, Sul1, Sul2, Sul3, tet(A), dfrA, floR, catB3, mphA, mphB | R  (>256) | S  (0.125) | S  (2) | R  (32) | R  (64) | S  (0.25) | S  (2) | S  (2) |
