# Supplementary material for: Genomic epidemiological analysis of mcr-1-harboring Escherichia coli collected from livestock settings in Vietnam
Source: Front Vet Sci. 2022 Oct 26;9:1034610. doi: 10.3389/fvets.2022.1034610 (PMC9643773; doi:10.3389/fvets.2022.1034610)
Supplement: Supplementary file 2 [file Table_2.DOCX]

Table S2. The occurrence of virulence-related genes in various phylogenetic groups

of MCRPEC strains

| **Presence of Virulence Genes** | **Phylogroups** | | | | **Total** | |
| --- | --- | --- | --- | --- | --- | --- |
|  | **Group A** | **Group B1** | **Group C** | **Group F** | **No. with Virulence Genes** | **%** |
| aslA | 34 | 0 | 1 | 2 | 37 | 74.0 |
| astA | 12 | 2 | 0 | 1 | 15 | 30.0 |
| chuS | 1 | 0 | 0 | 2 | 3 | 6.0 |
| chuT | 0 | 0 | 0 | 2 | 2 | 4.0 |
| chuU | 1 | 0 | 0 | 2 | 3 | 6.0 |
| chuV | 1 | 0 | 0 | 2 | 3 | 6.0 |
| chuW | 1 | 0 | 0 | 2 | 3 | 6.0 |
| chuY | 1 | 0 | 0 | 2 | 3 | 6.0 |
| csgB | 35 | 9 | 1 | 2 | 47 | 94.0 |
| csgD | 35 | 9 | 1 | 2 | 47 | 94.0 |
| csgF | 36 | 9 | 1 | 2 | 48 | 96.0 |
| csgG | 37 | 9 | 1 | 2 | 49 | 98.0 |
| entA | 37 | 9 | 1 | 2 | 49 | 98.0 |
| entB | 36 | 9 | 1 | 2 | 48 | 96.0 |
| entC | 36 | 9 | 1 | 2 | 48 | 96.0 |
| entD | 36 | 9 | 1 | 2 | 48 | 96.0 |
| entE | 37 | 9 | 1 | 2 | 49 | 98.0 |
| entF | 36 | 9 | 1 | 2 | 48 | 96.0 |
| entS | 37 | 9 | 1 | 2 | 49 | 98.0 |
| fdeC | 20 | 8 | 0 | 2 | 30 | 60.0 |
| fepA | 36 | 9 | 1 | 2 | 48 | 96.0 |
| fepB | 36 | 9 | 1 | 2 | 48 | 96.0 |
| fepC | 37 | 9 | 1 | 2 | 49 | 98.0 |
| fepD | 37 | 9 | 1 | 2 | 49 | 98.0 |
| fepG | 36 | 9 | 1 | 2 | 48 | 96.0 |
| fes | 37 | 9 | 1 | 2 | 49 | 98.0 |
| fimA | 25 | 6 | 1 | 2 | 34 | 68.0 |
| fimB | 22 | 4 | 1 | 2 | 29 | 58.0 |
| fimC | 27 | 6 | 1 | 2 | 36 | 72.0 |
| fimD | 27 | 6 | 1 | 2 | 36 | 72.0 |
| fimE | 22 | 5 | 1 | 2 | 30 | 60.0 |
| fimF | 32 | 8 | 1 | 2 | 43 | 86.0 |
| fimG | 34 | 8 | 1 | 2 | 45 | 90.0 |
| fimH | 34 | 8 | 1 | 2 | 45 | 90.0 |
| fimI | 26 | 6 | 1 | 2 | 35 | 70.0 |
| fyuA | 3 | 1 | 0 | 0 | 4 | 8.0 |
| gspC | 17 | 6 | 1 | 2 | 26 | 52.0 |
| gspD | 18 | 6 | 1 | 2 | 27 | 54.0 |
| gspE | 19 | 6 | 1 | 2 | 28 | 56.0 |
| gspF | 19 | 6 | 1 | 2 | 28 | 56.0 |
| gspG | 19 | 6 | 1 | 2 | 28 | 56.0 |
| gspH | 20 | 7 | 1 | 2 | 30 | 60.0 |
| gspI | 20 | 7 | 1 | 2 | 30 | 60.0 |
| gspJ | 19 | 7 | 1 | 2 | 29 | 58.0 |
| gspK | 18 | 7 | 1 | 2 | 28 | 56.0 |
| gspL | 19 | 7 | 1 | 2 | 29 | 58.0 |
| gspM | 22 | 7 | 1 | 2 | 32 | 64.0 |
| iroN | 1 | 0 | 0 | 0 | 1 | 2.0 |
| iutA | 0 | 0 | 0 | 1 | 1 | 2.0 |
| kpsD | 3 | 0 | 0 | 2 | 5 | 10.0 |
| kpsM | 3 | 0 | 0 | 1 | 4 | 8.0 |
| ompA | 38 | 9 | 1 | 2 | 50 | 100.0 |
| papC | 1 | 0 | 0 | 0 | 1 | 2.0 |
| yagV/ecpE | 27 | 7 | 0 | 2 | 36 | 72.0 |
| yagW/ecpD | 26 | 7 | 0 | 2 | 35 | 70.0 |
| yagX/ecpC | 27 | 7 | 0 | 2 | 36 | 72.0 |
| yagY/ecpB | 29 | 7 | 0 | 2 | 38 | 76.0 |
| yagZ/ecpA | 28 | 7 | 0 | 2 | 37 | 74.0 |
| ykgK/ecpR | 27 | 7 | 0 | 1 | 35 | 70.0 |
